# Biochemical and structural characterization of alanine racemase from *Bacillus anthracis *(Ames)

**DOI:** 10.1186/1472-6807-9-53

**Published:** 2009-08-20

**Authors:** Rafael M Couñago, Milya Davlieva, Ulrich Strych, Ryan E Hill, Kurt L Krause

**Affiliations:** 1 Department of Biochemistry, University of Otago, Dunedin, New Zealand; 2 Department of Biochemistry Rice University, Houston, TX, USA; 3 Department of Biology and Biochemistry, University of Houston, Houston, TX, USA

## Abstract

**Background:**

*Bacillus anthracis *is the causative agent of anthrax and a potential bioterrorism threat. Here we report the biochemical and structural characterization of *B. anthracis *(Ames) alanine racemase (Alr_*Bax*_), an essential enzyme in prokaryotes and a target for antimicrobial drug development. We also compare the native Alr_*Bax *_structure to a recently reported structure of the same enzyme obtained through reductive lysine methylation.

**Results:**

*B. anthracis *has two open reading frames encoding for putative alanine racemases. We show that only one, *dal1*, is able to complement a D-alanine auxotrophic strain of *E. coli*. Purified Dal1, which we term Alr_*Bax*_, is shown to be a dimer in solution by dynamic light scattering and has a V_max _for racemization (L- to D-alanine) of 101 U/mg. The crystal structure of unmodified Alr_*Bax *_is reported here to 1.95 Å resolution. Despite the overall similarity of the fold to other alanine racemases, Alr_*Bax *_makes use of a chloride ion to position key active site residues for catalysis, a feature not yet observed for this enzyme in other species. Crystal contacts are more extensive in the methylated structure compared to the unmethylated structure.

**Conclusion:**

The chloride ion in Alr_*Bax *_is functioning effectively as a carbamylated lysine making it an integral and unique part of this structure. Despite differences in space group and crystal form, the two Alr_*Bax *_structures are very similar, supporting the case that reductive methylation is a valid rescue strategy for proteins recalcitrant to crystallization, and does not, in this case, result in artifacts in the tertiary structure.

## Background

*Bacillus anthracis *is a soil-dwelling, spore-forming, Gram-positive bacterium that is the causative agent of the zoonotic disease anthrax. Although the disease is most common in wild and domestic mammals, it can also occur in humans when exposed to infected animals or living spores [1]. The severity of anthrax in humans depends on the route of infection. Inhalation of *B. anthracis *spores can lead to the most severe form of the disease, historically associated with a case-fatality rate as high as 85% [2,3]. The high mortality rate, the existence of a respiratory route of infection and the great resistance of its spores has made *B. anthracis *the subject of biological warfare research programs in many countries for over 60 years [4]. The United States Centers for Disease Control and Prevention (CDC) has classified anthrax as a category A bioterrorism agent, posing the greatest possible threat to public health and with the ability to spread across large areas [5]. In 2001, the Ames strain of *B. anthracis *was used in a series of bioterrorist attacks that resulted in five fatalities and cost billions of dollars to the US economy [6,7]. As *B. anthracis *spores are resilient, remaining viable and infective for many years, efforts to decontaminate affected facilities are time-consuming and costly. Therefore, it would be of significant importance to public health and security to develop new strategies aimed at containing *B. anthracis *spores upon their release into the environment.

Alanine racemase (EC 5.1.1.1) is an essential enzyme in prokaryotes. The enzyme utilizes a pyridoxal 5'-phosphate (PLP) cofactor to catalyze the racemization of L-alanine to D-alanine, an essential component of the peptidoglycan layer in bacterial cell walls. The lack of alanine racemase function in eukaryotes has made this enzyme an attractive target for antimicrobial drug development [8,9]. In *B. anthracis*, the gene coding for alanine racemase, *dal1*, is one of only four genes up-regulated during sporulation [10]. The *dal1 *gene product (Alr_*Bax*_) is found on the spores' outermost layer [10] and addition of alanine racemase inhibitors has been shown to promote germination of *B. anthracis*' spores [11] while endogenous production of D-alanine, mediated by alanine racemase, inhibits germination [12]. Triggering the premature germination of *B. anthracis *spores by spraying alanine racemase inhibitors on affected areas may therefore be a strategy to speed decontamination efforts and reduce the risk of infection in humans. Further, Alr_*Bax *_has recently been reported as an immunodominant protein in a proteomic analysis of the *B. anthracis *spore induced immunome [13,14]. Given the importance of three-dimensional information in structure-aided inhibitor design [15-17] and its growing role in vaccine development [18-20], structural studies on Alr_*Bax *_are crucial to both of these goals.

The first structural studies of alanine racemases, which were performed on the enzyme isolated from *Geobacillus *(then *Bacillus*)*stearothermophilus*, revealed a homodimeric enzyme with each monomer consisting of an α/β barrel domain at the N-terminus and a C-terminal domain composed mainly of β strands. The active site is located at the interface of the α/β barrel and β domain near a PLP cofactor forming an internal aldimine linkage to a lysine residue [21,22]. Structural studies performed in the presence of substrate analogs have identified the residues involved in catalysis and shed light onto the enzyme's catalytic mechanism [23]. Moreover, structures of *G. stearothermophilus *alanine racemase with the covalent inhibitors alanine phosphonate and D-cycloserine (DCS) have shown that enzyme inhibition is due to the covalent link of these compounds to the PLP cofactor and helped explain their non-specific inhibition of eukaryotic PLP-containing enzymes [24,25].

Alanine racemase structures from the human pathogens *Pseudomonas aeruginosa *and *Mycobacterium tuberculosis *have been solved [23] and both revealed further insights into the enzyme's catalytic site that may lead to identification of new, more specific inhibitors. The high-resolution (1.45 Å) structure of alanine racemase (DadX) from *P. aeruginosa *showed evidence for an external aldimine linkage of an unanticipated guest substrate in the active site [23], while the *M. tuberculosis *structure revealed that the narrow entryway to the enzyme's active site is composed of highly conserved residues distributed in layers beginning at the PLP site [26]. The structure of the DCS-producing *Streptomyces lavendulae *has also been determined [27]. *S. lavendulae *can grow in the presence of DCS, and the structural basis for the slower inhibition rate of DCS on *S. lavendulae *Alr has been attributed to the enzyme's larger and more rigid active site [27].

Here we report the cloning and characterization of the two genes, *dal1 *and *dal2*, from the *B. anthracis *genome with sequence similarities to other bacterial alanine racemase genes. Although expression of *dal2 *in a heterologous system failed, we have successfully expressed and purified the gene product of *dal1*, which we term Alr_*Bax*_, and performed its kinetic and structural characterization.

Recently another group has reported that *B. anthracis *alanine racemase crystallization required reductive methylation [28]. Interestingly we have not found this to be the case. However, the availability of both structures, one with and one without methylation, allows for a careful comparison to be performed. Reductive methylation has been employed previously to obtain atomic structures for proteins recalcitrant to crystallization [29-33]. Due to its reported successes, this method is becoming more utilized [28,34,35]. Nevertheless, there may be concerns as to how methylation impacts protein structure. Our analyses of both structures suggest that despite differences in space group and crystal lattice, reductive methylation does not significantly alter the structure of alanine racemase from *B. anthracis*.

## Results and Discussion

### Sequence analysis of the *Bacillus *Dal proteins

**Figure 1:**
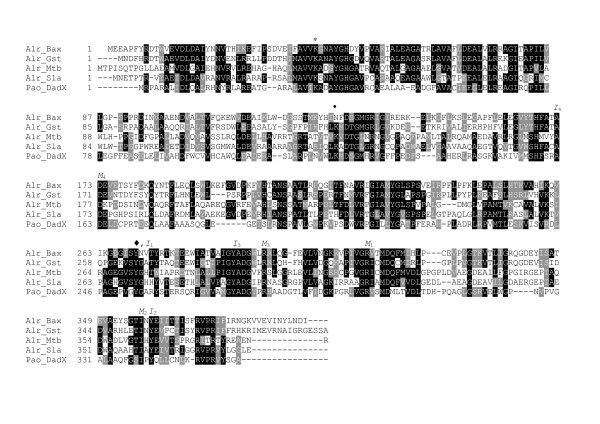
**Structure-based alignment of alanine racemasesfrom *B. anthracis *(AlrBax), *G. stearothermophilus *(AlrGst), *M. tuberculosis *(AlrMtb), *S. lavendulae *(AlrSla) and *P. aeruginosa *(DadXPao)**. The initial alignment was performed using EXPRESSO (3DCoffee) [63] and adjusted manually upon inspection of the superimposed structures. An asterisk marks the location of the Lys residue bound to PLP, the diamond marks the location of the Tyr residue that functions as the second base in the racemase reaction, a bullet denotes the location of the carbamylated lysine found in other alanine racemase structures and replaced by a chloride ion on Alr_*Bax*_. I and M denote residues found in the middle or the inner layer of the active site entryway along with their position in the entryway.

The sequences for both *dal1 *and *dal2 *genes amplified in our laboratory from *B. anthracis *(Ames) genomic DNA are 100% identical to those previously deposited in GeneBank (*dal1 *GeneID: 1087014 and *dal2 *GeneID: 30262102) [36]. The protein sequences encoded by *dal1 *and *dal2 *both contain the characteristic motifs expected for members of the alanine racemase family, such as a PLP-binding site near the N-terminus, the two key conserved catalytic amino acid residues Lys41 (Alr_*Bax *_numbering) and Tyr270, and a set of conserved residues making up the entrance corridor to the alanine racemase active site [26] (Figure 1). The gene product of *dal1*, which we term Alr_*Bax*_, is identical to the alanine racemase protein previously associated with germination in *B. anthracis *spores [37] and shares 57% amino acid identity with Alr_*Gst*_. Dal2, on the other hand, shows 41% sequence identity to Alr_*Bax *_and 40% identity to Alr_*Gst*_.

### Complementation analysis

In order to confirm that both *dal1 *and *dal2 *genes encode functional alanine racemases we expressed these genes in a D-alanine auxotrophic strain of *E. coli*, MB2795 [38]. Expression of the *dal1 *gene from *B. anthracis *or *dadX *from *P. aeruginosa *fully restored the wild-type phenotype. Cells transformed with pET28-TEV failed to grow, as did those transformed with the *B. anthracis dal2 *gene (data not shown).

### Overexpression, purification and biochemical characterization of Dal proteins

We used strain BL21(DE3), pLysS of *E. coli *to express *dal1 *and *dal2 *recombinant gene products. While *dal1 *was expressed successfully, the expression of *dal2 *failed repeatedly, even when conditions such as temperature, IPTG concentration, or strain background were changed (data not shown). Sequencing of the plasmid construct revealed no obvious errors, and our expression system has been successfully used for numerous proteins in the past. We have no conclusive explanation for our inability to express *dal2 *at measurable levels in *E. coli*. While the *orf *appears to encode an alanine racemase enzyme, it clearly is not expressed in the T7 overexpression system, possibly also explaining the lack of complementation observed in our earlier experiments. Given that, to our knowledge, there are no reports characterizing *dal2 *in the literature, we are led to believe that this gene is not usually expressed in its homologous host. As overproduction of Dal2_*Bax *_failed, all subsequent work was performed with *dal1 *to yield a product, which we term Alr_*Bax*_. Alr_*Bax *_was purified to homogeneity and displayed a single peak on molecular sieve chromatography.

Previous studies have suggested Alr_*Bax *_might exist partly as a tetramer in solution [34]. We have used dynamic light scattering (DLS) to determine that Alr_*Bax *_has a hydrodynamic radius of 3.7 nm, corresponding to a molecular weight of 93 kDa. As the calculated molecular weight of Alr_*Bax *_is 43.7 kDa, this enzyme is unambiguously a dimer (ca. molecular weight 87.4 kDa) in solution under the conditions of this experiment. These measurements were made on a monodisperse solution of Alr_*Bax *_in which 99.9% of the mass was accounted for by the single peak at 3.7 nm.

We find that purified Alr_*Bax *_has a *K*_*m *_for D-alanine of 2.8 mM and a *V*_*max *_of 101 U mg^-1^, where one unit was defined as the amount of enzyme that catalyzed racemization of 1 μmol of substrate per minute. These kinetic parameters for racemization of L- to D-alanine of Alr_*Bax *_fall in the range of what has been observed before for other bacterial alanine racemases [38-40]. Interestingly, despite the high identity levels observed for residues in the active site of Alr_*Bax *_and Alr_*Gst *_(Figure 1), the *V*_*max *_for the anthrax enzyme is one order of magnitude lower than the one reported for the *G. stearothermophilus *enzyme and closer to that observed for alanine racemases isolated from other pathogenic organisms. Our kinetic characterization reinforces previous observations that there is a very wide dynamic range in kinetic constants for alanine racemase, despite the sequence and structural similarities of their active sites.

### Description of the Overall Structure of Alr_*Bax *_from B. anthracis

**Figure 2:**
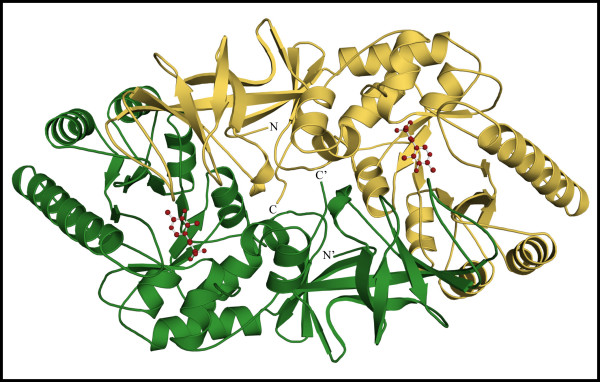
**Ribbon representation of the dimer of the alanine racemase from *B. anthracis***. The PLP co-factor is shown as a ball and stick model. Monomers are shown in different colors. N and C indicate the position of the C- and N-termini of one monomer; primed letters denote the termini for the second monomer.

Consistent with other alanine racemases, the tertiary structure of Alr_*Bax *_is a homodimer formed by head-to-tail-association of two monomers (Figure 2). Each monomer is crystallographically distinct in this crystal form (Table 1), but the two monomers have very similar folds. The rms difference obtained for their C_α _atoms after least-squares superposition is 0.22 Å. Alr_*Bax *_monomers consist of two structurally distinct domains. Residues in the N-terminus (16–245) fold into an eight-stranded α/β barrel, while the C-terminal residues (246–389) and the first 15 N-terminal amino acids are part of a predominantly β-structure. The homodimer displays two active sites, formed by residues from the N-terminus of one monomer and residues from the C-terminus of the other monomer. The PLP cofactor forms a covalent bond to Lys41 and points at the center of the α/β barrel. As previously observed for Alr_*Gst *_[22], extra density was present in the active of Alr_*Bax*_, which we model here as a molecule of acetate.

**Table 1:** Data-collection and structure-refinement statistics.

Data collection	
Space group	P2_1_

Unit-cell parameters	a = 49.62 Å, b = 141.27 Å, c = 60.12 Å α = γ = 90.00°, β = 103.11°

Observations	150,355 (12,217)

Unique reflections	53,396 (5,695)

Resolution (Å)	32.79–1.95 (2.06–1.95)

Completeness (%)	91.3 (67.1)

R_merge _(%)	2.9 (15.6)

Mean ((I)/σ(I))	22.2 (5.4)

Redundancy	2.8 (2.1)

Refinement statistics	

Resolution (Å)	32.79–1.95 (2.01–1.95)

Reflections	51,760 (2,518)

Total atoms	6,436

R_work _(%)	16.0 (19.40)

R_free _(%) (for 1627 reflections)	20.1 (23.4)

Average B factor (Å^2^)	

main chain	34.7

side chain	36.6

PLP	29.7

RMS deviations	

bond length (Å)	0.017

bond angles (deg.)	1.46

no. of residues	772

no. of protein atoms	6038

no. of PLP atoms	30

no. of acetate atoms	8

no. of water atoms	358

no. of chloride ions	2

Values in parentheses are for the highest resolution shell.

### Structural comparisons of Alr_*Bax *_with closely related enzymes

Below we compare Alr_*Bax *_to the highly active Alr from the non-pathogenic bacterium *G. stearothermophilus *(Alr_*Gst*_) as well as the less active Alrs from pathogenic bacteria *P. aeruginosa *(DadX_*Pao*_) and *M. tuberculosis *(Alr_*Mtb*_). We also compare Alr_*Bax *_to the Alr from the DCS-producing bacteria *S. lavendulae *(Alr_*Sla*_). These enzymes share between 26 and 57% sequence identity (Figure 1 and Table 2). The crystal structure of native Alr_*Bax *_reveals some structural features that may be responsible for its slower catalytic rate and suggests regions that might be targeted in designing inhibitors of this enzyme.

**Table 2:** Average r.m.s.d. (Å) between the Cα atoms of Alr_Bax _and other Alrs

Alanine Racemase	Whole monomers^a^	N-terminal domains^b^	C-terminal domains^c^	Active site^d^
Alr_*Gst*_	1.10 (57%)	1.07 (57%)	0.60 (59%)	0.45 (73%)
Alr_*Sla*_	1.71 (37%)	1.66 (36%)	1.12 (39%)	0.73 (51%)
Alr_*Mtb*_	1.84 (39%)	1.64 (36%)	1.11 (40%)	0.83 (44%)
DadX_*Pao*_	2.30 (26%)	1.82 (26%)	1.43 (32%)	0.81 (41%)

Numbers in parenthesis denote sequence identity with Alr_*Bax*_. ^a^Calculated using monomer A. ^b^Calculated using residues 4–245. ^c^Calculated using residues 246–389. ^d^Calculated using residues 39–45, 63–67, 84–88, 103–107, 127–140, 163–171, 203–210, 221–228, 356–363 from monomer A and 268–271 and 314–319 from monomer B.

As noted, the *B. anthracis *Alr_*Bax *_secondary structure and general fold closely resembles that seen for other alanine racemases [23]. However, there are a few small topological differences between the structures of Alr_*Gst *_and Alr_*Bax*_. Alr_*Bax *_is five residues longer than Alr_*Gst*_; three of the five extra residues in Alr_*Bax *_extend Helix 8 by one turn; while the remaining two extra residues locate to the very N-terminus of Alr_*Bax*_. Helix 8 does not take part in the enzyme's active site nor does it make intermonomer contacts, therefore, we do not anticipate this secondary structure to play a critical role in Alr_*Bax *_function.

Least-squares superposition of C_α _atoms from N- and C-terminal domains from Alr_*Gst*_, Alr_*Sla*_, Alr_*Mtb *_and DadX_*Pao *_to the equivalent domains in Alr_*Bax *_reveals average rms differences ranging from 1.10 to 2.30 Å. The rms differences correlate with sequence identity levels (Table 2). Superposition of the N-terminal domains of Alr_*Bax *_and Alr_*Gst *_reveals significant C_α _deviations (≥ 1.8 Å) for residues in three loops (residues 121–125, between H6 and S6; residues 198–202, between H8 and S8; residues 215–219, between H9 and S9) and residues 148–158 on H7. These regions all locate to the protein surface and have no reported role in homodimer formation or substrate binding and catalysis. On the other hand, superposition of the C-terminal domains of Alr_*Bax *_and Alr_*Gst *_shows no regions with C_α _rms differences greater than 1.4 Å.

### Alr_*Bax *_and Alr_*Gst *_have a similar hinge angle between N- and C-terminal domains

**Figure 3:**
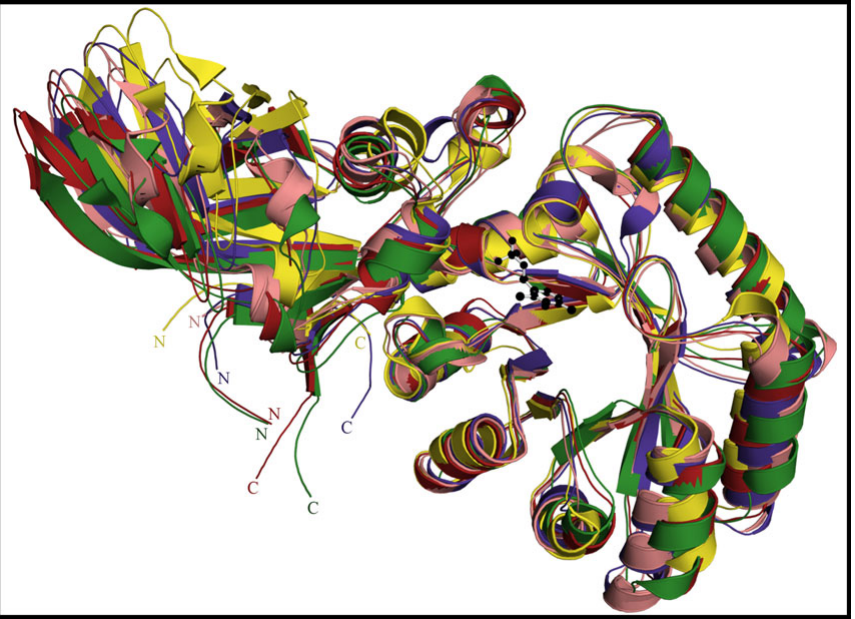
**Differences in hinge angle between various alanine racemases**. Structural alignment of the N-terminal domain α/β barrel from *B. anthracis *(green), *G. stearothermophilus *(red), *M. tuberculosis *(pink), *S. lavendulae *(blue) and *P. aeruginosa *(yellow) shows that the hinge angle between N- and C-terminal domains can vary. N- and C-termini are labeled N and C and colored according to their respective structures. The PLP cofactor for Alr_*Bax *_is shown as a ball and stick model in black.

The overall rms differences among various bacterial alanine racemases (Table 2) suggest that despite their topological similarity there are notable structural differences between their individual domains. It has been reported previously that the hinge angle between N- and C-terminal domains varies among different alanine racemases [23]. It is due to this difference that monomers from different alanine racemases cannot be optimally superimposed onto each other as a whole. While a single hinge angle comparison was sufficient for the pair-wise analysis previously given, the inclusion of more racemase structures has let us to consider a new system of hinge angle description. In this system, following superposition of the N-terminal domains, we define the shift in the C-terminal domains of one racemase compared to another through two rotation angles. One is measured relative to a plane parallel and one relative to a plane perpendicular to the PLP ring (Figure 3). The hinge rotations relative to the N-terminal domain may be relevant for enzyme activity as it could influence the position of the second catalytic residue, Tyr270' (primed numbers denote residues found in the second monomer). For the planes parallel and perpendicular to the PLP ring the rotation for the C_α _atom of Tyr270' from Alr_*Bax *_compared to the structurally equivalent atom in Alr_*Gst*_, Alr_*Mtb*_, Alr_*Sla *_and DadX_*Pao *_is 0.4/2.7°, 3.9/8.2°, 8.7/2.3° and 10.3/5.9°, respectively (Figure 3). Given that Alr_*Gst *_and Alr_*Bax *_have the most similar hinge angles we have compared these two structures with the other Alrs in order to establish which regions are responsible for the hinge angles in various Alrs.

**Figure 4:**
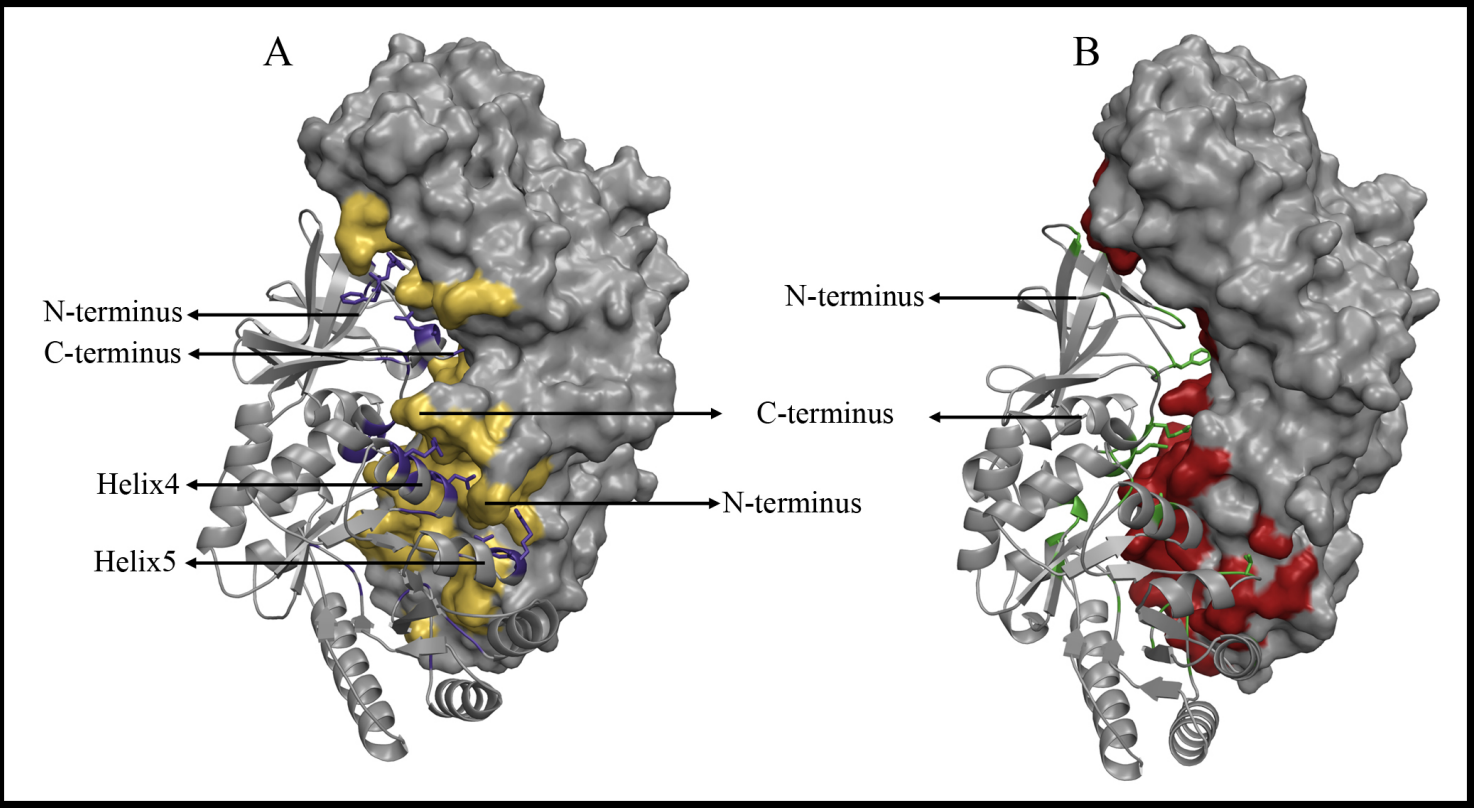
**Molecular surface for one monomer of the alanine racemases from *B. anthracis *(A) and *S. lavendulae *(B) docked into the ribbon diagram of the opposite monomer**. Alr_*Bax *_has extended intermonomer contacts at its C- and N-termini that are not present for other alanine racemases like the one from *S. lavendulae*. Position of residues taking part in intermonomer contacts are shown in purple and yellow for Alr_*Bax *_and green and red for Alr_*Sla*_. For Alr_*Bax*_, residues on Helices 4 and 5 and on N- and C- termini that make intermonomer polar contacts are shown as sticks. Equivalent residues taking part in intermonomer contacts are also shown as sticks for Alr_*Sla*_. Positions for the N- and C- termini, Helix 4 and Helix 5 are indicated by arrows.

Differences in hinge angles between various Alrs were first reported by us for Alr_*Gst *_and DadX_*Pao *_and attributed to specific polar interactions present only in Alr_*Gst*_. These interactions are mediated by polar side chains of residues Asp68 and Arg89 of the first Alr_*Gst *_monomer and the polar side chain of Asn379 and main chain O from Phe4 and main chain N from His5 of the second monomer [23]. Here in Alr_*Bax*_, we find equivalent polar interactions facilitated by structurally analogous residues; Asp70 and Arg93 of the first Alr_*Bax *_monomer and Phe6, Tyr7 and Asn384 of the second monomer. An additional interaction takes place between Asp77 from Helix 4 and Leu386 and Ile389 in Alr_*Bax*_. Therefore, the polar contacts that have been proposed to mediate the hinge angle in Alr_*Gst *_are also present in Alr_*Bax*_, and as noted above these two structures have the most similar hinge angles. On the other hand, these polar contacts are not seen for Alr_*Mtb*_, Alr_*Sla *_and DadX_*Pao*_. Figure 4 shows that the side chains of residues in Alr_*Bax *_taking part in intra-monomer interactions make extensive contacts with residues on the second monomer, when compared with structurally equivalent residues in Alr_*Sla*_. Alr_*Mtb*_, Alr_*Sla *_and DadX_*Pao *_are shorter than Alr_*Gst *_and lack structurally equivalent residues to the C- and N-terminus of the longer enzymes (Figure 1). For example, Alr_*Sla *_has no analogous residue to Asn384 in Alr_*Bax*_as the peptide chain is only 380 residues long. Moreover, the structurally equivalent residue for the polar arginine is Gly89. It is not surprising that the hinge angles of these three deviate most from Alr_*Gst*_.

This analysis notwithstanding, the relevance of the hinge angle to enzyme catalysis in alanine racemase remains unclear. Although the *V*_*max *_values reported for all alanine racemases studied to date vary by over three orders of magnitude, it is not straightforward to attribute these differences to hinge angle. Differences in hinge angles certainly alter the relative orientation of the two active sites in the dimer, but affect very little the geometry of each active site as indicated in Table 2. Further, the *V*_*max *_for Alr_*Gst *_and Alr_*Bax *_enzymes varies by more than 10 fold, despite having similar hinge angles. Altering the hinge angle of this enzyme experimentally through mutation or cassette swapping may resolve this issue.

### Intermonomer contacts and surface area

The dimer interface in alanine racemase is an important area for structural analysis. Only the dimeric form of the enzyme is catalytically active [41]. Therefore, interface residues are critical in forming a functional active site. Certainly the interface functions to correctly position the second catalytic tyrosine residue from the opposite monomer on top of the active site. In addition, both monomers contribute to the overall composition of the alanine entryway and binding pocket. Loss of interface contacts would alter this arrangement and could be used as a strategy to inhibit Alr activity. Disruption of dimer interfaces is becoming more common and has been successfully used recently for drug targets in HIV and HCV [42-44].

**Figure 5:**
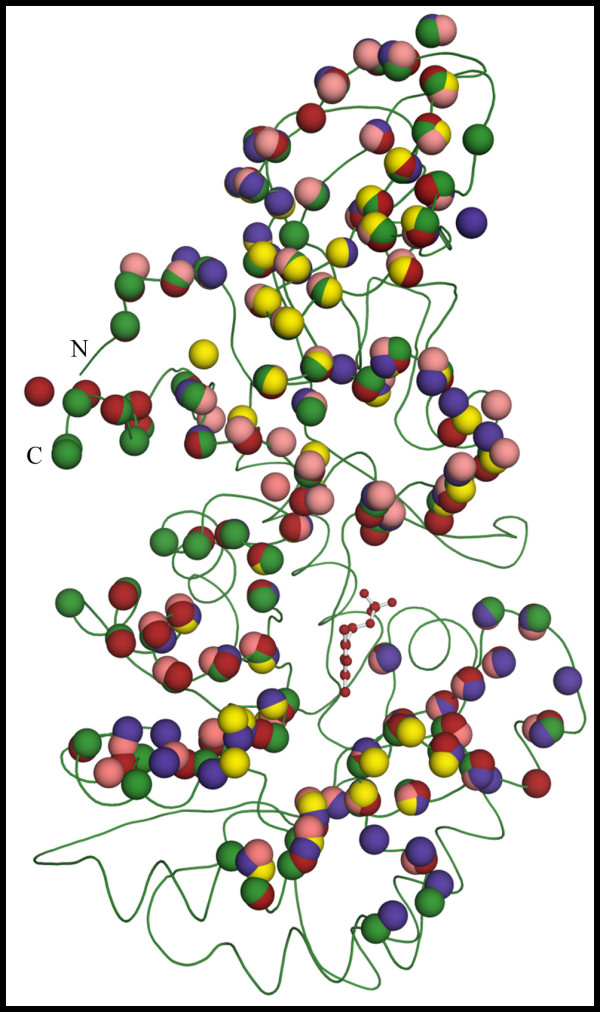
**Position of residues taking part in intermonomer contacts is highly conserved among various Alrs, despite differences in hinge angles**. Following a structural alignment of the N-terminal domains of various Alrs, the position for the Cα atoms from residues that take part in intermonomer contacts and are in the N-terminal domain (shown as colored spheres) was plotted on the main chain representation of Alr_*Bax *_(shown in green). Likewise, the position for the Cα atoms from residues that take part in intermonomer contacts and are at the C-terminal domain (shown as colored spheres) were plotted on the main chain representation of Alr_*Bax *_after a structural alignment of the C-terminal domains of various Alrs. Residues are colored according to the legend on figure 3. The PLP cofactor from Alr_*Bax *_is shown as a ball and stick model. N and C indicate the position of the C- and N-termini in the monomer.

Despite large differences in hinge angles, the location of interface residues in various Alrs is very similar (Figure 5). In Figure 5, the Cα atoms for residues taking part in intermonomer contacts in various Alrs are shown as colored spheres. The positions for the various spheres were obtained following two independent structural alignments, one using only atoms from the N-terminal domain and the other using only atoms from the C-terminal domain, and then plotted onto a ribbon diagram of Alr_*Bax*_. If the position of residues taking part in intermonomer contacts is conserved among various Alrs, we would expect the colored spheres to form tight clusters, containing superimposed red, green, blue, yellow and pink spheres. Indeed, as shown in Figure 5, most of the intermonomer contacts from various Alrs are found in clusters and thus are conserved among various Alrs. It is important to keep in mind that the number of residues taking part in intermonomer contacts varies among the analyzed Alrs. For Alr_*Bax*_, 94 of its 389 residues take part in intermonomer contacts and both N- and C-terminal domains contribute an almost equal number (44 and 50, respectively) of residues to the interface. The total number of residues in the interface of Alr_*Gst*_, Alr_*Mtb*_, Alr_*Sla *_and DadX_*Pao *_is slightly smaller than in Alr_*Bax*_. Nevertheless, for all analyzed structures, both domains contribute almost equally to the monomer-monomer interface.

At its dimer interface, the Alr_*Bax *_structure displays a larger surface area and higher number of polar interactions than Alr_*Gst*_, Alr_*Sla *_and DadX_*Pao *_(Table 3). Not surprisingly, most of the additional buried surface area observed for Alr_*Bax *_results from the interactions involving N- and C-terminal residues described in the hinge angle analysis above. If residues from the N-terminus (4–10) and C-terminus (383–389) of Alr_*Bax *_are excluded from the calculation, the intermonomer surface area of Alr_*Bax *_is reduced from 3,500 to 2,500 Å^2^, making it similar to the values found for Alr_*Sla *_and DadX_*Pao *_(~2700 Å^2^) (Table 3).

**Table 3:** Intermonomer interactions for alanine racemases

alanine racemase	Intermonomer surface area (Å^2^)	hydrogen bonds	salt bridges
Alr_*Bax*_	3536	38	9
Alr_*Gst*_	3083	31	12
Alr_*Sla*_	2798	20	7
DadX_*Pao*_	2788	14	7

### Alr_*Bax *_PLP-binding and active site

**Figure 6:**
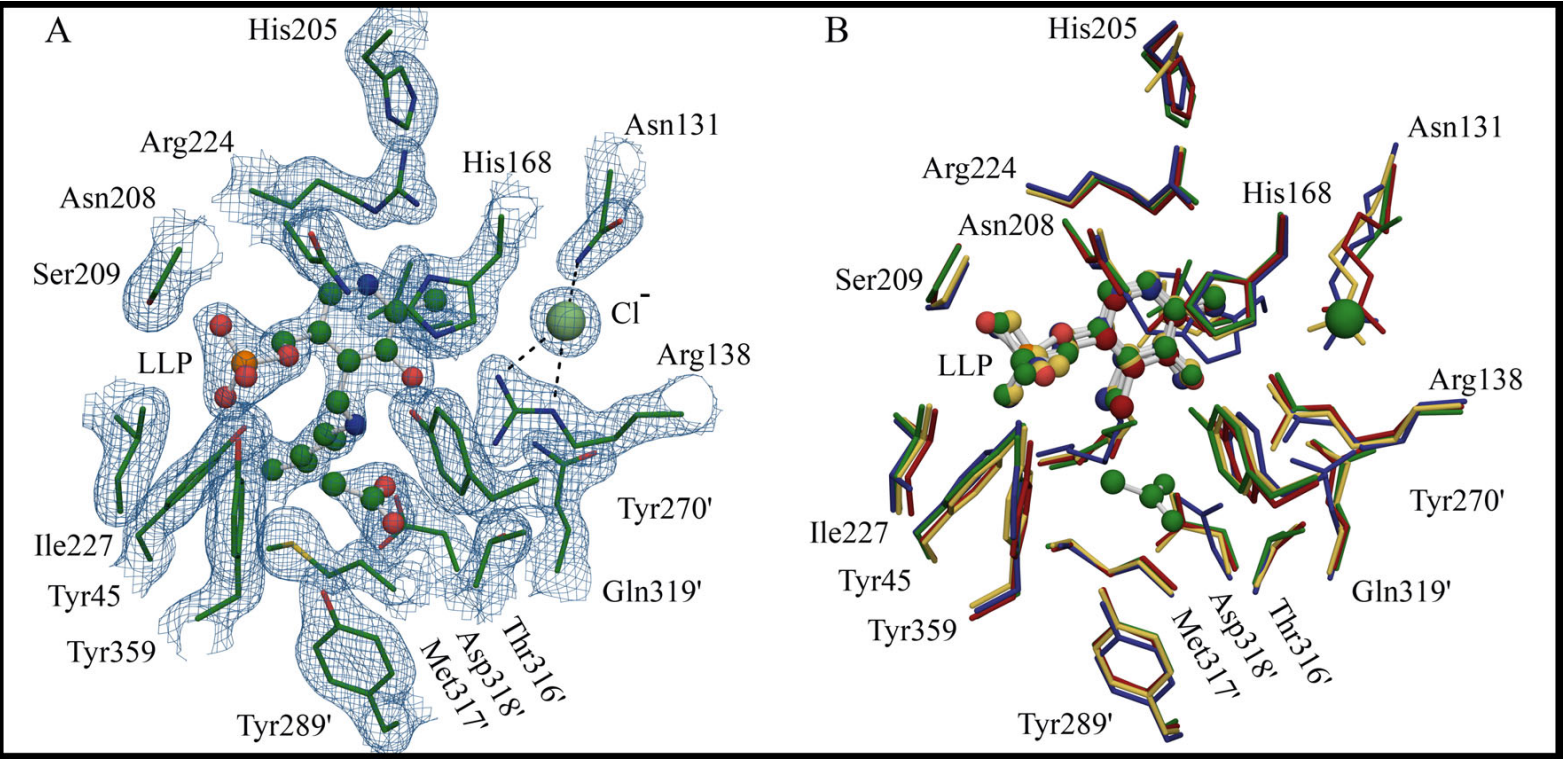
**Organization of the active site residues in *B. anthracis *Alr is facilitated by a chloride ion**. (A) Electron density map (contoured at 1.5σ in the final refined 2F_o_-F_c _map) showing details of the active site for Alr_*Bax*_. (B) Structural alignment of residues making the active site of various Alrs (TB structure was not included). For all available Alr structures, Arg138 makes polar contacts to the PLP and, possibly, to the substrate. In Alr_*Bax *_this arginine residue is positioned in the active site by a chloride ion (Cl^-^). Polar contacts between the chloride ion and Asn-131 and Arg-138 are shown in Panel A by dashes. For all other alanine racemase structures available to date the equivalent interactions are mediated by a carbamylated lysine (shown in Panel B). Residues in the active site of various Alrs are shown as a stick model. In Panel A, the acetate molecule and the modified lysine residue (LLP) are depicted as ball and stick models; carbons are colored in green, nitrogen in blue, oxygen in red, phosphate in orange, sulfur in yellow and the chloride ion is depicted as a light green sphere. In Panel B residues are shown as stick model and are colored according to the legend on figure 3; the PLP cofactors are shown as ball and stick models. In both panels, primed numbers denote residues from the second monomer.

As observed for other Alrs, the active site of Alr_*Bax *_is formed by residues from both monomers, with the two catalytic bases Lys41 and Tyr270' found in different monomers. In the Alr_*Bax *_structure, Lys41 is seen covalently linked to the PLP cofactor. As was observed for one of the Alr_*Gst *_structures (1sft) [22], we have identified extra density in the active site of Alr_*Bax*_, which we have modeled as a molecule of acetate. Acetate, which was present in our crystallization solution, is an inhibitor of Alr [22] and its carboxylate group is thought to bind the enzyme active site in the same way the carboxylate group from alanine is expected to do [22]. The oxygen atoms from the acetate molecule in our model are within hydrogen bonding distance to the side chain oxygen from Tyr289', the main chain nitrogen from Met317' and, perhaps more importantly, to the side chain nitrogen atom from the catalytic Lys41 residue (Figure 6).

The identity and position of active site residues is strongly conserved among various Alrs (Table 2). As a result, the hydrogen bonding network found for the PLP molecule in the active site of Alr_*Bax *_is similar to the one observed for other Alrs. In Alr_*Bax*_, side chain atoms from Tyr45, Arg138, Arg24, His168, Ser209 and Tyr359 establish hydrogen bonds to atoms in the PLP cofactor (Figure 6). These residues are strictly conserved for Alr_*Gst*_, Alr_*Mtb*_, Alr_*Sla *_and DadX_*Pao *_and have similar orientations in the PLP-binding site of their respective enzymes. The PLP in Alr_*Bax *_also hydrogen bonds with main chain atoms from Ser209, Gly226 and Ile227. The first two of these residues is strictly conserved in Alr_*Gst*_, Alr_*Mtb*_, Alr_*Sla *_and DadX_*Pao*_. The third would be as well but in Alr_*Sla*_, the Ile227 is replaced by a leucine residue. Perhaps a more significant difference is the presence in Alr_*Sla *_and Alr_*Mtb *_of a tryptophan residue in place of Alr_*Bax *_Leu87. A tryptophan residue at this position is one of the differences found between the active sites of the slower enzymes from *M. tuberculosis *and *S. lavendulae *and the faster Alr from *G. stearothermophilus*. In Alr_*Sla *_and Alr_*Mtb*_, the Nε atom of this tryptophan makes a water-mediated hydrogen bond to O3 from PLP. Although this extra interaction may have a role in catalysis it does not seem to reduce the size of the Alr_*Sla *_and Alr_*Mtb *_active sites as the loop that harbors this tryptophan residue is shifted away (~2.1 Å) from the PLP cofactor when compared to the same loop in Alr_*Bax*_. Mutagenesis studies could thus be performed in order to evaluate the impact of this tryptophan residue for enzyme catalysis.

One striking difference in the active site involves Asn131, which in other alanine racemases is generally a carbamylated lysine that participates in a hydrogen bond with the residue homologous to Arg138. In Alr_*Bax*_, however, we note a prominent chloride ion that is located near Arg138 in the active site (Figure 6). This chloride ion has not been described in Alr structures from other species and it was originally modeled by us as a water molecule. However, the resulting low B-factor (~10 Å^2^) and its hexa-coordination with three water molecules and atoms Nδ2 from Asn131 and Nε and Nη2 from Arg138 suggested the presence of a chloride ion. Notably, there is no chloride present in the crystallization buffer and we can only assume that the enzyme binds so tightly to this halide that it is carried over from the enzyme's purification. The chloride ion is also observed on the Alr_*Bax *_structure obtained following lysine reductive methylation [30]. The presence of a chloride ion in two independent structures reinforces the idea that this ion plays an important structural role in Alr_*Bax*_. Other Alrs have a negative charge at the same position, but the charge has always been from a carbamylated lysine residue (Figure 6). In the Alr_*Mtb *_structure a carbamylated lysine was not noted but the side chain density for this lysine was poor. Like the chloride ion in Alr_*Bax*_, the carbamyl group found in other Alrs hydrogen bonds with Nε and Nη2 from the active site arginine (Arg138 in Alr_*Bax*_), thus positioning this residue in the active site. The general conservation of the modified lysine residue among various Alrs and its role in positioning the active site arginine indicates that the presence of a negative charge at this position is critical for enzyme catalysis. As Alr_*Bax *_lacks the conserved lysine residue necessary for carbamylation it has apparently drafted a chloride ion to fill the same role for this species. It is open to speculation whether the addition of chloride chelators like SPQ (6-methoxy-N-(3-sulfopropyl)-quinolinium) would affect the enzyme activity and whether it might be possible to design specific inhibitors for Alr_*Bax *_based on this unique interaction.

In Alr_*Bax *_in addition to the interactions facilitated by the chloride ion, Arg138 is further positioned by the side chain oxygen of Thr316'. Further, an alignment of 105 Alrs, having between 24% to 99% sequence identity to Alr_*Bax*_, revealed that the presence of an asparagine at the equivalent position to Asn131 in Alr_*Bax *_is always accompanied by the presence of a threonine residue equivalent to Thr316' (data not shown) suggesting that this interaction with Arg138 would be a conserved feature of alanine racemases with active site structural chlorides. Sequences of Alrs that contain a lysine in position 131 almost always have an accompanying serine or a cysteine residue in position equivalent to Alr_*Bax *_Thr316'. In the case of Alr_*Pao *_this serine is involved in an equivalent active site arginine interaction. The exception to this latter observation is Alr_*Sla *_which has an alanine at this position. It is important to note that there is not really a specific chloride-binding motif as the residues that interact with Cl^- ^in Alr_*Bax *_are the same that interact with the carbamylated lysine in the other structures.

### The active site entryway of Alr_*Bax*_

Residues from loops in the α/β barrel domain of one monomer and residues from the C-terminal domain of the second monomer make up an entryway to the active site and the PLP binding site. The active site entryway of Alr has been previously divided in inner, middle and outer layers, starting from the PLP binding pocket and moving towards the protein surface [26]. Residues in the inner and middle layers show strong conservation among various Alrs [26]. For Alr_*Bax*_, residues Tyr270', Tyr359, Tyr289' and Ala172 constitute the inner layer, while residues Arg314', Ile357, Arg295' and Asp173 make up the middle layer. These residues are absolutely conserved between Alr_*Bax *_and Alr_*Gst*_, Alr_*Mtb*_, Alr_*Sla *_and DadX_*Pao*_. The outer layer for the active site entryway of various Alrs displays less conservation, but in this region Alr_*Bax *_contains an Asn271' while Alr_*Gst*_, Alr_*Mtb*_, Alr_*Sla *_and DadX_*Pao *_contain a glycine. As a result of this substitution, the entryway is somewhat more restricted than the ones observed for other alanine racemases. Finally, for Alr_*Bax *_a conserved pair of acidic residues (Asp-Glu) is found at positions 173 and 174, which are located in the middle and outer layers of the entryway. Identical residues are found in the same position for Alr_*Gst*_, Alr_*Sla *_and DadX_*Pao*_, but for Alr_*Mtb*_, a much slower alanine racemase, these two residues are (Asp-Lys). This site has recently been shown to be important catalytically, as making this Asp-Glu to Asp-Lys change at the same position in *E. coli *alanine racemase has been shown to significantly decrease its catalytic rate [45].

### Structural comparison of native and reductively methylated alanine racemases from *B. anthracis*

Recently, the structure of Alr_*Bax *_after reductive methylation of its lysine residues (Alr_*BaxRM*_) has been reported [28]. In that report, the unmodified protein failed to crystallize. Scientists at the Oxford Protein Production Facility (OPPF) and the York Structural Biology laboratory reported that extensive crystallization trials (approximately 800 conditions) with native Alr_*Bax *_proved unsuccessful and that reductive lysine methylation was essential for crystallization of the protein [34,46]. Based on data from mass spectroscopy and on the methylated crystal structure of Alr_*Bax*_, Au and colleagues concluded that the N terminus and 18 out of the 20 lysines in Alr_*Bax *_were methylated after the protein was treated with dimethyl-amine-borane complex and formaldehyde.

Reductive methylation modifies all free primary amines in a protein molecule (NH groups from lysine residues and the N terminus) to tertiary amines. This modification of lysine residues, especially those found on the protein surface, offers an opportunity to change a protein's crystallization properties and is a proven method to rescue proteins recalcitrant to crystallization [28-33,35,47]. However, there are few structural studies showing that reductive alkylation does not alter a protein's structure, especially of proteins that do not readily crystallize. One study [48] reporting on the effects of reductive lysine methylation on HEW lysozyme found that crystals were formed under different conditions and with a different crystalline lattice than observed for the unmodified enzyme. Nevertheless, the structures of both modified and unmodified enzymes showed no significant structural differences and their superimposed Cα atoms had an rms difference of only 0.4 Å [48]. The availability of native and modified structures for Alr_*Bax*_, therefore, offers another opportunity to evaluate the impact of reductive lysine methylation, this time on a protein more recalcitrant to crystallization.

In our hands, Alr_*Bax *_protein readily formed small crystals using commercially available crystallization screens. Notably our form contains eight additional residues at the C-terminus that remain following cleavage of a C-terminal His-tag using TEV protease. These residues are not involved in crystal contacts, but still could have an influence on crystallization. Our initial crystallization conditions required extensive fine-tuning, and the addition of the glutathione additive proved important for obtaining diffraction quality crystals. Moreover, finding the proper conditions for freezing Alr_*Bax *_crystals without compromising diffraction quality proved challenging. For simplicity's sake we have referred to this form of Alr_*Bax *_as unmethylated or native. Our review of the expression and purification protocols for both native and alkylated enzymes suggests that they were very similar. Also, modified and unmodified Alr_*Bax *_crystallize under similar conditions, despite a reported small reduction in the isoelectric point and the expected changes in the surface properties of Alr_*Bax *_[34]. Both proteins were crystallized in the presence of PEG (18% PEG 8000 for the native and 25% PEG 3350 for the modified protein), high salt concentrations (0.2 M sodium acetate for the native and 0.2 M magnesium chloride for the modified protein) and at the same pH, 6.5. Interestingly, the modified enzyme was crystallized at 60 mg/ml while the native structure was obtained from crystals grown at 15 mg/ml.

Despite similar crystallization conditions native and modified Alr_*Bax *_crystals show different crystalline lattices and solvent content. Native Alr_*Bax *_crystals are monoclinic with space group P2_1 _and unit cell parameters *a *= 49.6 Å, *b *= 141.3 Å, *c *= 60.1 Å and β = 103.11°. On the other hand, crystals for the methylated enzyme are orthorhombic in space group P2_1_2_1_2_1 _with cell dimensions of a = 57.6 Å b = 88.4 Å and c = 139.0 Å. Crystals for the modified enzyme display a lower solvent content (38% vs. 48%) and a higher packing density (1.99 Å^3^/Da vs. 2.35 Å^3^/Da) than native crystals.

### Crystal contacts comparison

**Figure 7:**
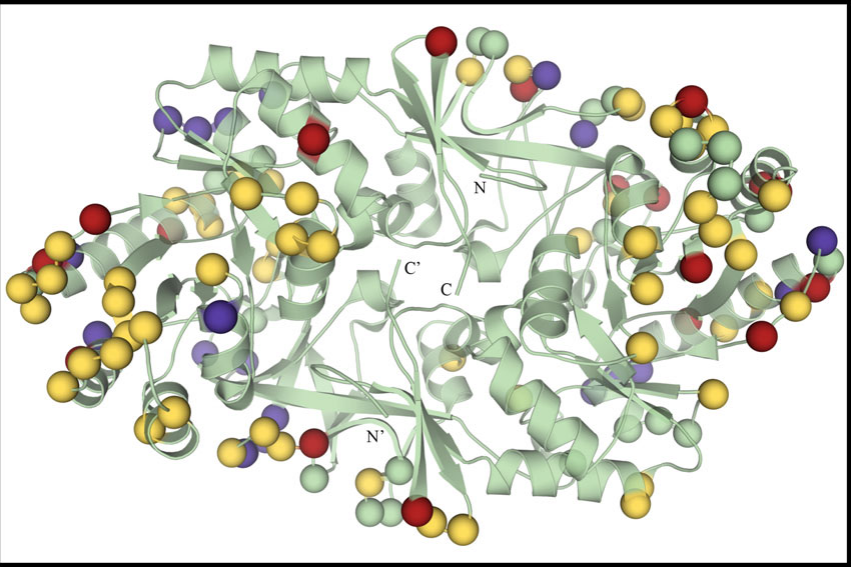
**Difference in crystal contacts following reductive methylation of secondary amines on Alr_*Bax*_**. The position of Cα atoms from residues making crystal contacts are shown as colored spheres superimposed onto the ribbon diagram of Alr_*Bax *_(shown in green). Methylated lysines involved in crystal contacts are shown in red; other residues involved in crystal contacts for the methylated structure only are shown in yellow. Residues implicated with crystal contacts for the native structure only are shown in blue. Residues found to make crystal contacts in both structures are shown in green. N and C indicate the position of the C- and N-termini of one monomer; primed letters denote the termini for the second monomer.

The total surface area found in crystal contacts for the reductively methylated enzyme is 1.7 times larger than that found for the native enzyme (1529.7 Å^2 ^vs. 918.4 Å^2^, respectively). Further, these contacts are often mediated by methylated lysine residues found at the protein surface (Figure 7). In monomer A from Alr_*BaxRM*_, 6 out of the 18 modified lysines contact protein atoms from both monomers in adjacent asymmetric units. For monomer B, 9 modified lysines engage in crystal contacts; contacting protein atoms in both monomers from symmetry related protein molecules. Interestingly, methylated lysine 202 from monomer A contacts the same residue from monomer B in a symmetry related molecule. In Figure 7, the location of the Cα atoms from residues taking part in crystal contacts for both the native and methylated structure is shown as colored spheres. Different colors were used for various categories of contacts. Yellow and red spheres are for contacts observed only in crystals of the methylated protein, while blue spheres are found only in the crystals of the native protein. Contacts found in both crystal forms are shown as green spheres. Figure 7 illustrates that crystals of the methylated Alr_*Bax *_contain more residues taking part in crystal contacts, and as noted above, modified lysine residues, shown as red spheres, make many of these crystal contacts. In this case of Alr_*Bax *_reductive methylation does change the protein surface in a way to promote the formation of a more extensive and apparently more ordered crystalline lattice than that found for the native crystals.

The surfaces of the modified and native Alr_*Bax *_crystals are also different in terms of metal and halide content. Four magnesium and three chloride ions were found on the surface of modified Alr_*Bax *_and take part in crystal contacts. For the native Alr_*Bax *_structure we did not identify any metal or halide ions at equivalent positions. Furthermore, the temperature factors for these surface ions are quite low, with four less than 20 Å^2^, and many are involved in extensive electrostatic interactions. Perhaps the presence of additional metal ions observed exclusively for the methylated crystal form of Alr_*Bax *_acts to compensate for the loss of positive charges at the protein surface.

Most importantly, reductive methylation did not alter the overall fold of Alr_*Bax*_. Structural alignment of methylated and native Alr_*Bax *_shows no significant difference in their overall structures. For the individual monomers the rms difference between their Cα atoms is just under 0.4 Å. Alignment of the active site residues from the two Alr_*Bax *_structures shows that reductive alkylation of the enzyme did not result in any significant changes in the position and hydrogen bond pattern of active site residues and the PLP co-factor. Moreover, the hinge angle between N- and C-terminal domains is very similar for both modified and unmodified Alr_*Bax*_. Thus, the hinge angles observed for Alr_*Bax *_are inherent to this particular enzyme and not an artifact of crystallization. As an aside, this observation makes a strong argument that the disparate hinge angles observed for other Alrs are not a consequence of divergent crystal packing.

Reductive methylation also did not significantly alter the dimer interface, which is found to be comparable between methylated and unmethylated structures (3600 Å^2 ^vs. 3500 Å^2^, respectively). For the modified structure, two methylated lysines contribute atoms from their methyl groups to the interface; Mly182 and Mly255. The corresponding lysines in the native structure are not considered to be part of the interface; Lys182 displays poor density and did not have its complete side chain modeled in the native structure and no atoms from Lys255 in one monomer are in contact distance to atoms in the other monomer.

Only two lysines escaped methylation in the modified crystal structure, Lys41 and Lys260 [46]. Lys41 is found covalently bound to the PLP co-factor. Thus its NH group is not a primary amine and is not surprising that this residue is unaffected by the reductive methylation protocol. Lys260 is the lysine residue least exposed to the solvent and it makes hydrogen bonds to Gly137 and Arg138 which, in turn, hydrogen bonds to the phenolic oxygen of the PLP cofactor and to the substrate (see above). These two residues are, therefore, involved in either a covalent bond or a strong polar interaction in the present structure and thus predictably escaped reductive methylation.

## Conclusion

In conclusion, we report the high-resolution crystal structure of alanine racemase from the *dal1 *gene of *B. anthracis *and characterize it kinetically and in an *E. coli *complementation system. This structure contains some unique features in its active site including a structural chloride atom. It shares a similar hinge angle to its close relative from *Geobacillus *and has an active site and topology much like other members of this family. Based on the results shown here the active site of Alr_*Bax *_is as accessible for inhibitor binding as other alanine racemases studied to date. Furthermore, it is very likely that alanine racemase inhibitors like D-cycloserine or alanine phosphonate will be effective as modulators of sporulation. Finally, as treatment of spores will take place in the environment and not internally, the problems associated with non-specific PLP inhibition ascribed to these inhibitors should not detract from their usefulness in bioremediation. We look forward to exploring more structural studies on these inhibitors as they become available.

## Methods

### Amplification and cloning of the *B. anthracis alr *genes

Two putative open reading frames, *dal1 *and *dal2*, for alanine racemase from *B. anthracis *were identified through sequence comparisons using the known alanine racemase sequence from *G. stearothermophilus *[22] as a probe against the *B. anthracis *genome deposited in GenBank [36]. Two sets of primers were used in PCR to amplify the two putative *alr *genes from genomic DNA of *B. anthracis *(Ames), dal1–5' (5'-GGG GCC ATG GAA GAA GCA CCA TTT TAT CGT G-3')/dal1–3' (5'-CCC CCT CGA GTA TAT CGT TCA AAT AAT TAA TTA C-3') and dal2–5' (5'-GGG GCA TAT GAG TTT GAA ATA TGG AAG AG-3')/dal2–3' (5' CCCCCTGCAGAATCCGTAGGTTTTAAGGAC 3'), resulting in amplicons of 1169 bp and 1175 bp, respectively. The PCR products were sequenced, inserted into a modified pET28 vector (pET28-TEV) containing a C-terminal His-tag and a TEV protease cleavage sequence, LEENLYFQ/SLQVEH_6 _and cloned in *E. coli *MB1547. (/) denotes the location of the cleavage site.

### Complementation analysis

Characterization of the two cloned genes continued with their transformation into the D-alanine auxotrophic *E. coli strain *MB2795 [38]. A plasmid encoding the cloned *P. aeruginosa *DadX alanine racemase, pMB1921 [49], was used as a positive control. Plasmid pET28-TEV without any inserts served as the negative control. Cells were grown on solid LB medium with and without D-alanine supplementation, and scored for colony growth after 16 h at 37°C as described previously [49].

### Dal1 overexpression and purification

Cultures of *E. coli *BL21(DE3), pLysS containing the pET28-TEV-dal expression plasmids were grown at 37°C in LB medium containing 100 μg/ml kanamycin and 30 μg/ml chloramphenicol to an OD_600 _of 0.8. Expression of recombinant proteins was induced by addition of 0.5 mM IPTG and carried at 30°C for 19 hours. Cells were harvested by centrifugation and the cell lysate was cleared and loaded onto a Hi Trap affinity (Ni^2+^) column (GE Healthcare Life Sciences). The column was washed and Alr_*Bax *_eluted with a stepwise imidazole gradient. The C-terminal 6xHis tag was removed by treatment with His-tagged TEV protease (1 mg TEV protease per 10 mg of protein for 16 hours at 4°C). Alr_*Bax *_without the 6xHis tag was purified from the reaction mixture using the same chromatography strategy described above. Following concentration, Alr_*Bax *_was loaded onto a Pharmacia Superdex 200 Preparative Grade column; sample purity was assessed by SDS-PAGE to be greater than 95%.

### Dynamic light scattering

Purified Alr_*Bax *_was dialyzed against 20 mM Tris pH 8.0. Protein samples (1 mg/ml) were centrifuged (10 min. at 14,000 rpm) and filtered using 0.02 μm Whatman Anotop filters prior to recording data. All measurements were made at 298 K using the DynaPro system according to the manufacturer's instructions (Wyatt Technology).

### Enzyme Kinetics and Crystallization

The kinetic parameters (*K*_*m *_and *V*_*max*_) for the racemization reaction (D- to L-alanine) catalyzed by Alr_*Bax *_were estimated using the spectrophotometric alanine racemase assay as described previously [40]. Alr_*Bax *_crystallization screening trials were performed using the vapor diffusion method with sitting drops (5 μl of protein at 15 mg/ml and 5 μl of mother liquor) in 24-well plates incubated at 4°C. Initial screens revealed thin needle crystals growing in 20% PEG 8000, 0.2 M sodium acetate, 0.1 M sodium cacodylate, pH 6.5 [50]. Crystals were optimized using streak-seeding with crushed crystals and further optimized using additive screening resulting in rectangular, deep yellow crystals suitable for data collection. The final crystallization condition was 18% PEG 8000, 0.2 M sodium acetate, 0.1 M sodium cacodylate, pH 6.5, 0.01 M GSH (L-glutathione reduced), 0.01 M GSSG (L-glutathione oxidized).

### Data Collection and Processing

Crystals were passed through cryoprotectant solutions consisting of 20.7% PEG 8000, 0.2 M sodium acetate, 0.1 M sodium cacodylate supplemented with 3, 6, 9, 12, 15 and 18% (v/v) ethylene glycol, mounted into a nylon loop and flash frozen in liquid nitrogen at 110 K. A native data set was collected at 110 K on a Micromax 007 HF rotating-anode X-ray generator equipped with a copper anode, Hi-res optics, an RAXIS IV++ image-plate detector (Rikagu) using a frame width of 0.5° and an exposure time of 600 s. Images were integrated using MOSFLM [51], processed with SCALA [52] and analyzed using programs from the CCP4 suite [53]. Data collection and processing statistics for the native data set can be found in Table 1. Alr_*Bax *_crystallized in space group P2_1 _with unit cell parameters a = 49.62 Å, b = 141.27 Å, c = 60.12 Å and β = 103.11. There is one Alr_*Bax *_dimer per asymmetric unit.

### Structure Determination and Refinement

Molecular replacement was carried out with MolRep [54] using the *G. stearothermophilus *Alr (PDB entry – 1SFT) atomic coordinates [22]. Molecular replacement was performed assuming two monomers per asymmetric unit as suggested by a Matthew's coefficient of 2.35 [55] and resulted in the proper orientation of the search model in the crystal lattice (R_fac _43.6%; score 0.699). The primary sequence of the search model was changed to that of Alr_*Bax *_using *Coot *[56]. All structural refinements (32.79 – 1.95 Å) were carried in Refmac5 [57] using standard restraints and were followed by visual inspection of protein models and density maps in *Coot*. Ten cycles of positional refinement, performed using NCS restraints, resulted in *R *and *R*_*free *_of 23.9 and 27.2%, respectively. Waters were added using the arp_water function on Refmac5, and when the active site density was clearly interpretable, PLP was added to both active sites. A further 10 cycles of positional and B_iso _refinements brought *R *and *R*_*free *_to 19.6 and 23.7%, respectively. Water molecules with B-factors higher than 55.0 Å^2 ^and electron density lower than 1.0 σ on a 2*F*_*obs *_– F_calc _map were then deleted.

### TLS Refinement

*B. anthracis *crystals displayed somewhat anisotropic x-ray diffraction and previous alanine racemase structures have shown indication of subdomain movement. This encouraged us to try TLS refinement [58]. TLS analyses were carried on with different domains of the protein acting as a rigid body. All models resulted in similar improvements in *R *and *R*_*free *_and in the end we adopted the most parsimonious one, which treated all protein atoms found in the asymmetric unit as a rigid body. After TLS refinement, the R and *R*_*free *_were 16.0 and 20.1% with root-mean-square deviations from ideality for bond lengths of 0.017 and angles of 1.46° (Table 1). As noted above, inclusion of the C-terminal His-tag has resulted in eight additional residues in our sequence. In the final map we attempted to build some of these residues into extra density at the C-terminus, but as we did not gain anything in terms of *R *or *R*_*free *_we have elected to leave out the extra residues from this region in the final structure. Structure factors and final atomic coordinates for Alr_*Bax *_have been deposited in the Protein Databank (PDB ID 3ha1).

### Structural comparisons

The structure of Alr_*Bax *_was compared to other closely related enzymes; their accession numbers are: 1sft – Alr_*Gst *_bound with acetate [22]; 1vfh – Alr_*Sla *_with no ligand [27]; 2vd8 – methylated Alr_*Bax *_[28], 1rcq – DadX_*Pao *_[23] and 1xfc – Alr_*Mtb *_[26]. Structural alignments were performed using SSM [59]. Interface surface area was calculated using PISA [60]. The number of polar contacts (hydrogen bonds and salt bridges) was determined using WHAT IF [61,62].

## List of Abbreviations

Alr: alanine racemase; Bax: *Bacillus anthracis*; DCS: D-cycloserine; Gst: *Geobacillus stearothermophilus*; Mtb: *Mycobacterium tuberculosis*; Pao: *Pseudomonas aeruginosa*; PLP: pyridoxal 5'-phosphate; rms: root mean square; Sla: *Streptomyces lavendulae*.

## Authors' contributions

RC performed research, helped draft manuscript, analyzed results, and prepared figures. US performed research, helped draft manuscript, analyzed results. MD performed research, helped draft manuscript, and analyzed results. RH helped analyze structure and helped prepare figures. KK designed research, analyzed results, helped draft manuscript. All authors read and approved the final manuscript.
